# Laparotomy during Pregnancy

**Published:** 1895-07

**Authors:** Virgil O. Hardon

**Affiliations:** Atlanta, Ga.


ATLANTA

### Medical and Surgical Journal.

VOL. XII. JULY, 1895. No. 5.

LUTHER B. GRANDY, M.D., MANAGING EDITOR. MILLER B. HUTCHINS, M.D., BUSINESS MANAGER.

### *ORIGINAL COMMUNICATIONS.*

---

### LAPAROTOMY DURING PREGNANCY.

BY VIRGIL O. HARDON, M.D.,

ATLANTA, GA.

The day is long since past when an abdominal section attracts attention by its novelty. Lists of "consecutive laparotomies" no longer possess interest or value except to him who reports them. Abdominal work has come to have a recognized place in surgery, its indications have been well established, and its *technique* varies in the hands of different operators only in minor and unimportant details. There are, however, a few points which still admit of profitable discussion, either from the rarity of the conditions involved or from the unsatisfactory results which have attended their treatment. Among these points may be classed the performance of laparotomy during pregnancy.

It is comparatively seldom that the surgeon is called upon to open the abdomen of a woman who is normally pregnant. When conditions present themselves which would justify such a procedure in the non-pregnant woman, the existence of pregnancy offers a complication which gives us pause and leads us to consider seriously whether the interests of the patient would not be better served by postponing the operation until after confinement. There is always present a fear of interruption of the process of gestation, involving the sacrifice of the life of the child in the effort to save that of the mother. Many cases therefore which would otherwise be legitimate subjects for abdominal section can no longer be so regarded when two lives are at stake instead of one. Urgent symptoms threatening one or the other of these lives constitute the sole indication for surgical interference under such circumstances.

The altered conditions imposed upon the pelvic organs by pregnancy give a new and entirely different aspect to many diseases. The physiological engorgement and the rapid structural changes which accompany pregnancy, give to such diseases a different clinical history, and must be taken into consideration in discussing the prognosis and the treatment. The rapid development of tissue, most notable in the womb, but present also in other organs, extends to new growths in those organs and causes a rapidity of development of pathological processes proportionate to the rapid development of normal tissues. This fact must always be borne in mind as a factor in the prognosis. From it results the general principle that delay in operation which would be justifiable in the non-pregnant woman would often be unsafe in one who is pregnant.

Another question to be seriously considered in such cases is the effect which the diseased condition will have upon the fetus *in utero*. Conditions which are comparatively benign in their early stages as regards the life of the mother may, by pressure upon the uterus, or by the irritation of their presence, threaten miscarriage, with its attendant dangers. The question then presents itself whether the life of the mother will be more seriously jeopardized by operation, or by allowing miscarriage to occur. An additional question to be considered, is whether the physician has a moral right to allow the fetus to perish if by a comparatively safe operation upon the mother its life can be saved. The interests of the fetus cannot rightly be ignored except in those cases where efforts for its preservation would greatly augment the danger to the mother. It is a fortunate fact that in most of the cases which present themselves, the interests of mother and of fetus run in parallel lines.

Still another point to be considered is the effect which the operation itself will have upon the continuance of pregnancy. When the condition is one which is threatening the life of the mother, this question can very properly be ignored. But when the life of the fetus alone is hanging in the balance, it is evident that to be justifiable, the operation must be one which offers a fair prospect of removing the obstacle which threatens its life. There are certain pelvic conditions which in their very nature must be excluded from this category. Operations which involve mutilation of the womb cannot be undertaken without compromising the safety of the child. But experience has proven that operations for the removal of the ovaries and tubes, and even of pedunculated subperitoneal fibromas are not incompatible with the continuance of pregnancy. It has been shown that such operations not only do not produce miscarriage, but that they have no effect upon the subsequent development of the fetus.

Probably the most frequent pathological condition that we are called upon to consider in this connection is that of fibroma of the uterus. It has been generally stated that the tendency of such growths is to induce sterility, and that when pregnancy does occur spontaneous abortion usually results. In a measure both of these statements are true, especially when the tumors are intrauterine or intramural. But many exceptions occur. It not infrequently happens that with the growth of the uterus as pregnancy advances, the tumors rise up out of the pelvis and symptoms caused by pressure do not occur. When the tumor springs from the side of the womb however and lies between it and the pelvic wall, the uterus may be subjected to such pressure as to produce serious trouble and call for active interference. In such cases it was formerly considered necessary to produce abortion; but as a result of the development of abdominal surgery, several operators have successfully attempted the removal of such tumors during pregnancy. The success which has attended their efforts renders the induction of abortion under such circumstances no longer justifiable. Charpentier states that it is subperitoneal fibroids with long pedicles that are most apt to diminish the chances of pregnancy going on to term.

Fortunately it is this variety which is most amenable to treatment by laparotomy and removal. Another point bearing upon the question is that the induction of abortion apart from its inherent dangers does not rid the patient of her tumor; hence, the same difficulty is liable to occur with each succeeding pregnancy, in addition to other dangers to which the patient is exposed by the presence of the tumor itself. From all these considerations a rational conclusion appears to be that where active interference has become necessary, removal of the tumor by laparotomy is the operation to be preferred as best subserving the conjoint interests of mother and child.

Ovarian cysts constitute another pathological condition which, when complicating pregnancy, calls for interference. The aspect of such cases differs from those in which fibromas are present on account of the less resistant character of the tumor and its ability to accommodate itself in shape to the growth of the womb. The grounds for interference are found either in the excessive pain produced by the pressure of the tumor, which is crowded against surrounding parts by the increased size of the womb, or in the apprehension of difficulty in the delivery of the child at full term. In these cases we have a resource at our command which in many instances obviates the necessity of removal of the cyst during pregnancy. If the cyst be of considerable size so that it rises above the pelvic brim and can be felt by palpation through the abdominal wall, or if it projects downward into Douglas's pouch so as to lie in immediate contact with the vaginal wall, it may be safely tapped and its contents withdrawn. In this way the pressure symptoms may, for a time, be gotten rid of. The tumor will, however, refill, and it will be necessary to repeat the tapping with ever increasing frequency. A constant drain upon the patient's strength will result, and before the completion of the full term of pregnancy an alarming degree of exhaustion may render an operation imperative. The increased danger of operating upon a patient in this exhausted condition makes it necessary to consider carefully at the outset the probability of successfully carrying the patient through her pregnancy under this plan of treatment, and the advisability of removing the tumor while she is in possession of her full strength.

Cases sometimes occur in which the tumor, though of sufficient size to produce pressure symptoms, is not large enough to be easily reached by tapping either through the abdominal wall or from the vagina. The pain in such cases is not amenable to treatment other than the free use of opiates. The deleterious effects of continuous suffering upon the general health, and the equally injurious effects of the habitual use of opium, render a more radical treatment advisable in many such cases. It often happens, therefore, that while a patient with a large cyst can be successfully carried through her pregnancy by repeated tappings, it becomes necessary to subject a patient with a much smaller cyst to an abdominal section for its removal.

The presence of pus in the fallopian tubes is another condition which may call for operation during pregnancy. From a theoretical point of view, a woman who is suffering from pyosalpinx will never become pregnant. There can be no doubt, however, that pyosalpinx is sometimes present in pregnant women. In such cases it is to be presumed that one tube was unaffected previous to pregnancy, and furnished the pathway for the descent of the ovum, or that the disease developed *in toto* after conception had taken place. The existence of pregnancy does not protect a woman from the untoward results of pus in the tubes. Recurrent attacks of peritonitis, with their attendant dangers to health and even to life, form the usual history in such cases. If the woman goes to full term under such unfavorable conditions, her confinement is attended with greatly increased danger, the puerperal period is prolonged and hazardous, and she rises from her bed, if she rises from it at all, a physical wreck. There can be no doubt that many cases of fatal infection during and after labor arise from the existence of pus in the tubes. In view of these facts, it would appear to be the part of wisdom to forestall the grave dangers of puerperal infection by subjecting the patient to the lesser danger of the removal of the suppurating tubes during her pregnancy. The same arguments which are now universally accepted for the removal of such diseased organs in the non-pregnant woman, apply with even greater force to their removal when complicating pregnancy.

There are, perhaps, other conditions in which the question of opening the abdomen of a pregnant woman may properly be considered. But those which have been presented are the most frequent ones, and others are of very exceptional occurrence and are to be viewed upon the same general principles. Operations for carcinoma of the pregnant womb and for ectopic pregnancy do not come within the scope of this paper.

The *technique* of laparotomy upon the pregnant woman differs in no material respect from that of ordinary operations. The principal precaution to be observed is the avoidance of unnecessary handling or manipulation of the uterus. The physiological engorgement of the pelvic organs renders hemorrhage more likely to occur, and every precaution must be taken to stop bleeding before the abdomen is closed. The ligation of the ovarian arteries in the removal of both ovaries and tubes does not affect in any degree the continuance of pregnancy or the development of the fetus. This is readily understood when we consider the anatomical arrangement of the arteries which furnish the blood supply to the womb. The ovarian and uterine arteries are supplemental to each other, and if the circulation of blood through either be cut off, the other will take its place and the uterine circulation will not be interfered with. For this reason there is also no danger of imperfect involution of the womb after labor. The process goes on as perfectly as under normal circumstances. In closing the abdomen it is necessary to take special care that the edges of the incision be so united as to secure a firm union. The stretching of the abdominal wall as pregnancy advances renders the recent cicatrix liable to give way, with the production of ventral hernia. The different layers of the parietes should be brought into exact opposition, with especial care that the muscular tissue be fully included in the sutures. The drainage tube should be avoided if possible, as its use leaves a weak point in the line of union. A firm abdominal binder should be worn until after confinement to lessen the traction along the line of the incision.

I shall report two cases in which I have opened the abdomen during pregnancy.

CASE 1. L. A., married, aged twenty-six years. Has always had robust health. In April, 1890, became pregnant for the first time. In the following August she began to suffer severe uterine pains accompanied by slight hemorrhages from the womb. These symptoms led her to seek medical advice. When first seen by me, September 18, 1890, she presented the usual symptoms of pregnancy at four months. But by external palpation the enlarged womb could be felt to be pushed over into the left iliac region, while the right iliac region was occupied by a round, immovable tumor, apparently separate from the womb. By bimanual examination it was found that the tumor lay between the womb and the pelvic wall, that it was solid and attached to the womb by a thick pedicle. A diagnosis was made of a pedunculated subperitoneal fibroid, attached to the right side of the uterus, and impacted between the uterus and the pelvic wall. As the symptoms indicated a threatened miscarriage, and as the patient was exceedingly anxious that the child should be saved, it was decided to remove the tumor by laparotomy. The operation was performed September 25, 1890. Present, Drs. Harris and Kennedy. The abdomen was opened in the median line. Examination by the hand in the cavity confirmed the diagnosis. The tumor, which was of the size of an orange, was found to be free from adhesions, and by the exercise of a considerable degree of force was dislodged from the pelvis and brought up into the abdominal incision. It was found to be attached to the right side of the uterus by a pedicle as large as a man's wrist. The pedicle was tied in three portions by interlocking silk ligatures, which effectually controlled hemorrhage, and after being divided was dropped back into the abdomen. The abdomen was closed in the usual manner without drainage. The patient made an uninterrupted recovery, her temperature at no time reaching 100°. Without further symptoms of miscarriage she went on to the full term of her pregnancy, and on the 10th of March, 1891, was safely delivered by me of a living female child weighing ten and a quarter pounds.

CASE 2. Mrs. J. J. N., white, aged twenty-four, mother of two children. Has always enjoyed good health. Conceived in September, 1894. About a month after conception she began to suffer with severe pains in both ovarian regions, which increased until she was confined to her bed and was obliged to be kept constantly under the influence of opiates. Her general health became very much impaired, and her digestive powers failed until she was scarcely able to assimilate any food. On the 25th of November I examined her, and found both ovaries enlarged and excessively tender. Six days later the abdomen was opened and the ovaries removed. Both contained cysts, one as large as an English walnut, the other as large as a hen's egg. The patient made an uninterrupted recovery, and has suffered no pain since the operation up to present time. She has gained strength and flesh rapidly, and is able to digest and assimilate a normal amount of food. She is able to attend to all her social and domestic duties, and her pregnancy has gone on without interruption.

